# Effect of chitosan chewing gum on reducing serum phosphorus in hemodialysis patients: a multi-center, randomized, double-blind, placebo-controlled trial

**DOI:** 10.1186/1471-2369-15-98

**Published:** 2014-06-25

**Authors:** Tadao Akizawa, Yoshinari Tsuruta, Yoichi Okada, Yoshihiro Miyauchi, Akio Suda, Hiroshi Kasahara, Nobuhiro Sasaki, Yoshitaka Maeda, Takako Suzuki, Noriaki Matsui, Jun Niwayama, Toshiaki Suzuki, Hideaki Hara, Yasushi Asano, Sadao Komemushi, Masafumi Fukagawa

**Affiliations:** 1 Showa University School of Medicine, Tokyo, Japan; 2 Meiyo Clinic, Toyohashi, Japan; 3 Maruko General Hospital, Ueda, Matsumoto, Japan; 4 Asahi General Hospital, Asahi, Chiba, Japan; 5 Suda Clinic, Tokyo, Japan; 6 Japanese Red Cross Suwa Hospital, Suwa, Nagano, Japan; 7 Japanese Red Cross Koga Hospital, Koga, Ibaragi, Japan; 8 JA Toride Medical Center, Toride, Ibaragi, Japan; 9 Komagome Kyouritsu Clinic, Tokyo, Japan; 10 Tsuchiura Kyodo General Hospital, Tsuchiura, Ibaragi, Japan; 11 Sumiyoshi Clinic Hospital, Mito, Ibaragi, Japan; 12 Asagaya Suzuki Clinic, Tokyo, Japan; 13 Gifu Pharmaceutical University, Gifu, Japan; 14 Osaka City University Faculty of Engineering, Osaka, Japan; 15 Tokai University School of Medicine, Isehara, Japan

**Keywords:** Chewing gum, Chitosan, Clinical trial, Hemodialysis, Hyperphosphatemia, Phosphorus binders

## Abstract

**Background:**

HS219 (40 mg chitosan-loaded chewing gum) is designed to bind salivary phosphorus as an add-on to available phosphorus binders. We performed a randomized, placebo-controlled, double-blind study to evaluate the efficacy and safety of HS219 in hemodialysis (HD) patients with hyperphosphatemia as an add-on to phosphorus binders.

**Methods:**

Sixty-eight HD patients who were maintained on calcium carbonate (n = 33) or sevelamer hydrochloride (n = 35) were enrolled. The primary end point was a change in serum phosphorus levels. Secondary end points included changes in levels of salivary phosphorus, serum calcium, parathyroid hormone (PTH), and intact fibroblast growth factor (iFGF) 23.

**Results:**

Sixty-three patients chewed either HS219 (n = 35) or placebo (n = 28) for 30 min, three times a day, for 3 weeks. HS219 was well tolerated and safe. However, HS219 was not superior to placebo with additional reduction of serum phosphorus with respect to phosphorus binders at the end of the chewing period. There were no significant effects of HS219 on reduction of salivary phosphorus, serum calcium, iPTH, or iFGF23 levels.

**Conclusions:**

The chitosan-loaded chewing gum HS219 does not affect serum and salivary phosphorus levels in Japanese HD patients with hyperphosphatemia. Our findings do not support previous findings that 20 mg of chitosan-loaded chewing gum reduces serum and salivary phosphorus levels.

**Trail registration:**

ClinicalTrials.gov NCT01039428, 24 December, 2009.

## Background

Cardiovascular disease is the most common cause of death in patients with end-stage renal disease (ESRD) who are undergoing hemodialysis (HD) [1]. Increased levels of serum phosphorus in HD patients are associated with an increased incidence and mortality of cardiovascular diseases [2]. However, HD and restriction of dietary phosphorus result in insufficient control of serum phosphorus [3]. Oral phosphorus binders, such as calcium carbonate, sevelamer hydrochloride, lanthanum carbonate, and bixalomer, are currently used for the treatment of ESRD patients with hyperphosphatemia in Japan [4]. These phosphorus binders primarily trap dietary intake of phosphorus in the gut by an ion exchange reaction. Therefore, patients often have to take many phosphorus binder pills three times daily with meal, and this is associated with a risk of gastrointestinal symptoms and decrease adherence [5].

Notably, salivary phosphorus levels are higher than those in serum, particularly in patients with chronic kidney disease [6] and ESRD [7]. It is reported that the salivary flow rate in HD patients is similar to or less than that in healthy adults [8,9]. The daily saliva production ranges from 500 to 1500 mL [10]. Therefore, salivary phosphorus is an alternate source of phosphorus which should not be ignored, and could be a novel target for a novel phosphorus restriction.

Chitosan is a non-toxic natural fiber prepared by deacetylation of chitin from the shells of crustaceans, such as crabs and shrimps, and is used worldwide as a dietary supplement [11-13] and food additive [14]. The chemical structure of chitosan is a beta-1,4-linked polymer of D-glucosamine and its amino residue can stoichiometrically bind to phosphorus under physiological conditions.

HS219 is a three-layer chewing gum containing 40 mg of chitosan, which is designed to trap salivary phosphorus in the mouth. Savica et al. [15] reported that chewing a chitosan (20 mg)-loaded chewing gum for 60 min in fasting conditions twice a day for 2 weeks dramatically reduced salivary and serum phosphorus levels in HD patients with hyperphosphatemia as per serum phosphorus levels ≥6 mg/dl, despite treatment with sevelamer hydrochloride. However, this study was an open-label, single center clinical study in only 13 HD patients.

Therefore, we designed a multi-center, randomized, placebo-controlled, double-blind study to evaluate the efficacy and safety of HS219 when chewed for 30 min, 3 times a day for 3 weeks in HD patients with hyperphosphatemia whose serum phosphorus levels were not well controlled with either calcium carbonate or sevelamer hydrochloride.

## Methods

### Study design

This was a multi-center, randomized, double-blind, placebo-controlled, parallel-group, comparative study in Japan. ESRD patients with hyperphosphatemia who were poorly controlled by phosphorus binders were recruited from 11 study sites in Japan from December 2009 to June 2010. The protocol and informed consent form were reviewed and approved by one central Ethics Committee: Ethics Committee of the Japanese Red Cross Koga Hospital, Koga, Ibaragi, Japan and four site-based Ethics Committee/Institutional Review Boards: Ethics Committee of the Japanese Red Cross Suwa Hospital, Suwa, Nagano, Institutional Review Board of the Tsuchiura Kyodo General Hospital, Tsuchiura, Ibaragi, Ethics Committee of the Asahi General Hospital, Asahi, Chiba, and Ethics Committee of the JA Toride Medical Center, Toride, Ibaragi, Japan. All of the patients gave written informed consent before the initiation of any study-specific procedure. This study was carried out in accordance with the ethical principles of the Declaration of Helsinki and the Ethical Guidelines for Clinical Studies issued by the Ministry of Health, Labour and Welfare of Japan in 2003. This study was registered on December 24, 2009 as NCT01039428 at the ClinicalTrials.gov database.

### Patients

This study enrolled men and women with ESRD. They were ≥ 20 years of age and had been undergoing regular hemodialysis for three times a week for at least 12 weeks. The rate of urea reduction was controlled at > 60% for at least 4 weeks before they provided informed consent. They were maintained on either calcium carbonate or sevelamer hydrochloride alone with a stable regimen with respect to dose and frequency for at least 4 weeks before and throughout the study. The patients were required to undergo dietary phosphorus restriction throughout the study. The major inclusion criteria required a mean value of the latest three serum phosphorus levels of > 5.5 mg/dl and < 9.0 mg/dl, and a rate of salivary flow ≥ 1 g/2 min by the Saxon test [16]. The inclusion criteria of salivary flow rate was set based on a feasibility study conducted in Japanese HD patients (data not shown in this article) that indicated the number of the patients whose salivary flow rate > 2 g/2 min by the Saxon test were limited, and even a patient between 1 and 2 g/2 min were found to be able to chew the gum for 30 min. Patients who used active vitamin D derivatives and/or cinacalcet were required to have maintained a stable dosage and frequency for ≥ 4 weeks before and throughout the study. Patients were excluded from the study for the following reasons: they were being treated with blood purification therapy other than HD; they had a severe gastrointestinal motility disorder; they had any other clinically significant unusual medical conditions, such as malignancy or severe cardiovascular disorders; they had a history of chitosan, chitin, and/or shellfish allergy; and they were not able to chew the chewing gum. HD conditions including the dialysis time within 10% variation, blood flow rate within 10% variation, dialyzer, and dialysate were required to be maintained during the study.

### Interventions

The eligible patients were first stratified within each treatment arm by type of phosphorus binder (calcium carbonate or sevelamer hydrochloride), and were then randomized in a 1:1 ratio to receive HS219 or placebo chewing gum by computer-generated randomization with sequencing in blocks of four. Patients chewed either HS219 or placebo chewing gum three times a day while fasting (i.e., between meals) for 30 min for 3 weeks. A 3-week follow-up period for safety evaluation and efficacy recovery concluded the controlled study. All of the patients were treated and continued using the same single phosphorus binder (dosage and frequency) used at the time of screening, during and after the study. The patients’ adherence with the treatment, compliance with their phosphorus binder, and phosphorus-restricted diet throughout the study, including the follow-up period, were checked weekly by diary entries made by the patients. The patients were instructed how to chew the chewing gum before starting the study.

Blood was drawn every week (weeks 0, 1, 2, 3, 4, 5, and 6) before the first dialysis of the week (Monday or Tuesday) to determine serum phosphorus, calcium, and albumin levels. iPTH, whole PTH, and iFGF 23 were also measured at three points throughout the study as follows: before the treatment (week 0), at the end of the treatment (week 3), and at the end of the follow-up period (week 6). Whole saliva samples were collected using Salivette cotton (Sarstedt Ltd., Nümbrecht, Germany) before the first dialysis of the week at each patient’s visit. All participants were instructed not to eat, drink, or chew gum for at least 60 min before saliva collection.

Salivary phosphorus levels were determined by spectroscopic assay using a Biochain Phosphorus assay kit (CA, USA) at Gifu Pharmaceutical University. Serum phosphorus levels were analyzed at each institution. Serum levels of whole PTH and iPTH were analyzed by immunoradiometric assay and electrochemiluminescence immunoassay, respectively, at SRL Inc., Tokyo, Japan. iFGF 23 were determined using an FGF ELISA kit (Kainos Laboratories Inc., Tokyo, Japan) at a centralized laboratory.

### Chewing gum

HS219 is a three-layered tablet type of chewing gum, which contains 40 mg of food-grade medium-viscosity chitosan in the inner gum core layer. HS219 and its matching placebo chewing gum were provided by CM&D Pharma Limited, London, UK. Labeling and distribution were handled by Advance CRO Co., Ltd., Tokyo, Japan.

### Criteria for evaluation

The primary efficacy end point was changes in serum phosphorus levels from baseline to the end of the chewing period. Secondary end points included the ratio of patients whose reduction in serum phosphorus levels was ≥ 1.5 mg/dl from baseline to the end of the chewing period, the rate of patients whose serum phosphorus levels at the end of the chewing period were ≥ 3.5 mg/dl and < 5.5 mg/dl, and changes with time in levels of serum and salivary phosphorus, and serum calcium, phosphorus products (Ca × P), iPTH, whole PTH, and iFGF23. Adverse events, clinical laboratory assessments, and physical examination data were collected for evaluation of safety.

### Statistical analysis

The sample size in each treatment group was 26. Therefore, a two-group, one-sided U-test at a 2.5% significance level had 80% power to reject the null hypothesis that the difference in mean serum phosphorus levels between the HS219 and placebo groups at the end of treatment is <1.5 mg/dl. We assumed that there would be a reduction in 2.35 mg/dl of serum phosphorus with chewing HS219 according to Savica et al.’s study [15]. This was based on the assumption that the expected difference in the mean serum phosphorus levels between the chitosan group and the placebo group is 1.5 mg/dl, and the common standard deviation is 1.8 mg/dl [15]. To allow for a dropout rate of approximately 15% for the per-protocol analysis, a total sample size of 64 was appropriate.

Changes in serum phosphorus levels in each treatment group between baseline and at the end of chewing were compared using the Mann–Whitney U test, and changes were analyzed using analysis of covariance with treatment as a factor and the baseline value as the covariate. The last available (i.e., immediately after the chewing period) serum phosphorus level was used in the analysis of patients who discontinued treatment before the end of the experimental period. Data on the rates were compared using Fisher’s exact test and odds ratios. The 95% confidence interval (CI) for the estimated mean difference between mean levels of serum phosphorus, salivary phosphorus, serum calcium and serum phosphorus products (Ca × P) between the HS219 and placebo groups were determined in each week. Safety data were compared using Fisher’s exact test.

All analyses were performed using the “R Ver. 2.10.1” software package [17].

## Results

### Patients’ characteristics and disposition

Written informed consents were obtained from 71 patients, but two withdrew their consent and one failed to meet the salivary flow rate ≥ 1 g/2 min by the Saxon test before release of the randomization code. Sixty-eight patients (33 were using calcium carbonate and 35 were using sevelamer hydrochloride) were randomized, but five patients (n = 4, calcium carbonate; n = 1, sevelamer hydrochloride) withdrew from the study. Ineligibility of these patients was revealed after release of the randomization code: three patients failed to meet the eligibility criteria and two patients withdrew their consent. Sixty-three patients (n = 35, HS219; n = 28, placebo) chewed at least one tablet of chewing gum and were included in the intent-to-treat population for the analysis of efficacy and safety. Sixty-one patients (n = 33, HS219; n = 28, placebo) completed the 3-week treatment phase. One patients was not included because of withdrawal of consent and one was not included for failure to comply with the study protocol because of a change in dialyzer during the treatment phase.

**Figure 1:**
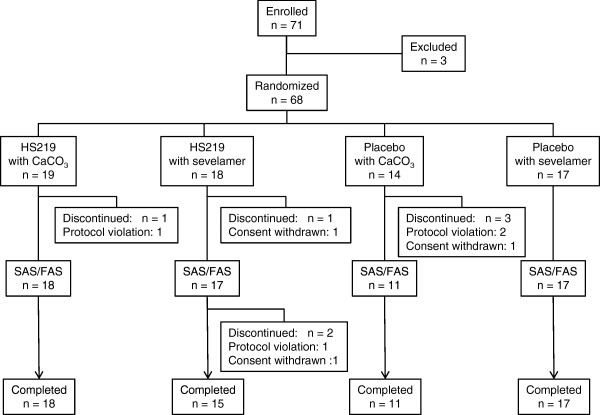
**Flow chart of patients enrolled in the study.** The following populations were defined. (i) The full analysis set (FAS) included a subset of patients who subsequently received chewing gum and had any of the variables for efficacy measured. (ii) The safety analysis set (SAF) included a subset of patients who received one or more doses of the chewing gum.

The demographic and clinical characteristics of the patient population were well balanced between the HS219 arm and placebo arm (Figure 1 and Table 1).

**Table 1:** Patient characteristics

	**HS219 n = 35**	**Placebo n = 28**	***p*-value**
Sex			
Male	24 (68.6%)	19 (67.9%)	1^†^
Female	11 (31.4%)	9 (32.1%)	
Age (years)	57 ± 11.	56 ± 11	0.585^‡^
	(34, 59, 79)	(30, 57.5, 76 )	
Weight before HD (kg)	59.7 ± 11.0	62.0 ± 16.4	0.861^‡^
	(40.8, 59.7, 86.7)	(39.3, 58.3, 120.4)	
Primary disease			
Chronic glomerulonephritis	20	19	0.204^‡^
Diabetic nephropathy	8	6	
Nephrosclerosis	2	2	
Polycystic kidney disease	0	1	
Others	5	0	
Duration of dialysis (year)	8.7 ± 6.8	8.7 ± 6.1	0.706^‡^
	(0.8, 7.3, 32.9)	(0.5, 8.2, 24.1)	
Hemodialysis time (hr)	4.2 ± 0.4	4.1 ± 0.3	0.978^‡^
	(3.5, 4, 5)	(3, 4, 5)	
Kt/V	1.43 ± 0.24	1.41 ± 0.22	0.718^‡^
	(1.09, 1.37, 2.07)	(1.10, 1.415, 1.87)	
Salivary flow rate (g/2 min)	3.91 ± 2.24	3.76 ± 1.69	0.986^‡^
	(1.02, 3.56, 10.3)	(1.46, 3.4, 7.42)	
Phosphate binder			
Calcium carbonate	18	11	0.447^‡^
Sevelamer	17	17	
Daily dose of phosphate binder			
Calcium carbonate (g/d)	2.4 ± 1.3	2.8 ± 1.5	0.548^‡^
	(1, 2, 6)	(1, 3, 6)	
Sevelamer hydrochloride (g/d)	2.7 ± 1.4	3.0 ± 2.1	0.952^‡^
	(0.75, 3, 5.25)	(0.75, 3, 7.5)	

Age, weight before HD, duration of dialysis, hemodialysis time, Kt/V, salivary flow rate, and daily dose of phosphate binder were presented by mean ± SD (min, median, max).

^†^Fisher’s exact test, ^‡^U-test.

### Efficacy of treatment

Changes in mean serum phosphorus levels from baseline to the end of treatment in the HS219 and placebo groups were -0.3 ± 1.2 mg/dl and -0.2 ± 1.2, respectively, with no significant difference between the two groups (p = 0.65) (Table 2).

**Table 2:** Changes in serum phosphorus level from baseline at week 3

**Treatment**		**Serum phosphorus Mean ± SD (mg/dl)**				
**Phosphate binder**		**n**	**Baseline**	**End of treatment**	**Change from baseline**	**U-test**
Overall	HS219	35	6.7 ± 1.0	6.4 ± 1.3	-0.3 ± 1.2	p = 0.652
	Placebo	28	6.5 ± 1.3	6.3 ± 1.3	-0.2 ± 1.2	
CaCO_3_	HS219	18	6.6 ± 1.0	6.1 ± 1.2	-0.4 ± 1.3	p = 0.799
	Placebo	11	6.3 ± 1.3	6.1 ± 1.0	-0.2 ± 0.9	
Sevelamer	HS219	17	6.7 ± 1.1	6.6 ± 1.5	-0.1 ± 1.0	p = 0.568
	Placebo	17	6.6 ± 1.3	6.4 ± 1.5	-0.2 ± 1.4	

The number of patients whose serum phosphorus level was reduced ≥ 1.5 mg/dl with chewing gum treatment was 7/35 (20%) in the HS219 group and 2/28 (7%) in the placebo group (p = 0.28). Lowering of serum phosphorus levels to achieve the targeted level of ≥ 3.5 mg/dl and < 5.5 mg/dl phosphorus (19) occurred in 8/31 (26%) patients in the HS219 group and in 2/23 (9%) patients in the placebo group (p = 0.16), respectively.

HS219 had no overall significant effect on changes with time in serum or salivary phosphorus, or serum calcium, Ca x P products, iPTH, or iFGF23 levels as secondary end points (Table 3).

**Table 3:** Salivary phosphorus, serum calcium, serum Ca x P, serum iPHT, serum whole PTH and iFGF23 levels at baseline and week 3

**Parameter**	**Period**	**HS219**		**Placebo**		**Mean difference**^**a**^	**95% CI**	**U test**
		**n**	**Mean ± SD**	**n**	**Mean ± SD**			
Serum phosphorus (mg/dl)	Baseline	35	6.7 ± 1.0	28	6.5 ± 1.3	0.2	-0.5 ~ 0.8	P = 0.677
	Week 3	32	6.4 ± 1.4	28	6.3 ± 1.3	0.1	-0.6 ~ 0.8	P = 0.900
Salivary phosphorus (mg/dl)	Baseline	35	24.0 ± 10.4	28	23.7 ± 8.9	0.2	-4.6 ~ 5.1	P = 0.964
	Week 3	32	22.3 ± 8.0	28	21.5 ± 9.0	0.8	-3.6 ~ 5.2	P = 0.656
Serum Ca (mg/dl)	Baseline	35	9.3 ± 0.7	28	9.2 ± 0.6	0.2	-0.1 ~ 0.5	P = 0.299
	Week 3	33	9.4 ± 0.7	28	9.1 ± 0.6	0.2	-0.1 ~ 0.6	P = 0.149
Serum Ca x P (mg^2^/dl^2^)	Baseline	35	62.0 ± 10.3	28	59.3 ± 11.1	2.7	-2.8 ~ 8.2	P = 0.490
	Week 3	33	59.9 ± 13.9	28	57.2 ± 11.3	2.7	-3.8 ~ 9.1	P = 0.520
iPTH (pg/ml)	Baseline	35	233 ± 148	28	233 ± 156	1	-77 ~ 78	P = 0.760
	Week 3	33	207 ± 141	28	251 ± 184	-45	-130 ~ 41	P = 0.511
whole PTH (pg/ml)	Baseline	35	133 ± 89	28	137 ± 97	-4	-51 ~ 44	P = 0.899
	Week 3	33	118 ± 92	28	140 ± 102	-22	-72 ~ 28	P = 0.511
iFGF23 (log_10_ pg/ml)	Baseline	35	4.1 ± 0.5	28	4.0 ± 0.6	0.1	-0.2 ~ 0.3	P = 0.736
	Week 3	33	4.0 ± 0.5	28	4.0 ± 0.5	0.0	-0.3 ~ 0.3	P = 0.960

^a^Mean difference between HS219 and placebo of group at baseline or week 3.

### Safety

The safety population included all of the patients who chewed at least one chewing gum (HS 219 or placebo) during the study period. Thirty-one adverse events were recorded in 21/63 patients. The overall incidence of adverse events was 13/35 (37%) in the HS219 group and 8/28 (29%) in the placebo group (p = 0.593). Adverse events not ruled out in relation to chewing gum were two in 63 patients; dizziness was observed with HS219 and loss of a tooth filling was observed with the placebo. No clinically significant abnormalities in laboratory values or vital signs were reported.

## Discussion

Salivary phosphorus is an alternative source of endogenous phosphorus, which is secreted into the mouth and then reabsorbed via the gut [6,7]. HS219 is a chitosan-loaded chewing gum, which is designed to bind salivary inorganic phosphorus directly in the mouth. HS219 was developed as a supplementary food for chronic kidney disease and ESRD patients with hyperphosphatemia who are treated with phosphorus binders.

Savica et al. reported an effect of chitosan (20 mg)-loaded chewing gum on serum and salivary phosphorus levels in HD patients with hyperphosphatemia who did not respond to a daily 3.2-4.8 g dose of sevelamer hydrochloride for 6 months and whose serum phosphorus levels were 7.6 ± 0.9 mg/dl [15]. They found that only 1-h chewing twice a day for 2 weeks dramatically reduced salivary and serum phosphorus levels by 55% (73.2 ± 19.2 to 33.2 ± 6.5 mg/dl, p < 0.00001) and 31% (7.6 ± 0.9 to 5.3 ± 0.9 mg/dl, p < 0.00001), respectively.

We aimed to confirm this surprising efficacy in Japanese HD patients by a randomized, placebo-controlled, double blind study. We considered that a daily 40 mg dose of chitosan for 2 weeks of treatment was too low to explain a reduction in salivary and serum phosphorus levels compared with the daily dose of calcium carbonate (3 g/day) and sevelamer hydrochloride (3–6 g/day). No information on the dose–response of chitosan was available in the literature [15]. Therefore, we investigated why chitosan-loaded chewing gum showed add-on effects to sevelamer hydrochloride based on quantitative analysis of Savica et al.’s report [15].

First, we increased the chitosan content in a chewing gum as much as possible. However, 40 mg of chitosan was the maximum amount loaded in a three-layered chewing gum. We increased the chewing times from two to three times a day, and designed the treatment period in our study for 3 weeks. The daily chitosan dose was 120 mg and the total amount of chitosan chewed in our study was 2520 mg. Our dose was 3 times higher than the daily dose and 4.5 times higher than the total dose compared with those in Savica et al.’s study (40 mg/day and 560 mg/study).

Chitosan is a polymer of glucosamine, which contains one amino residue. The molecular weight of this glucosamine unit is 161.2 (glucosamine - H_2_O) and the daily amount of 120 mg chitosan is 0.750 mmol. As well as an ion-exchange reaction, we expected ester formation between the two hydroxyl residues of glucosamine and phosphorus as additional phosphorus-binding capacities of chitosan [18]. The total number of phosphorus binding sites of the glucosamine unit is three and the total theoretical phosphorus binding capacity of 40 mg of chitosan is 2.25 (0.75 × 3) mmol. The pKa values of phosphate, 2.16, 7.21, and 12.32, indicate that mainly two ionic forms exist at pH 7.4. Therefore, the actual binding capacity under physiological conditions should be less than that calculated above. However, even if 2.25 mmol of chitosan would participates in binding stoichiometrically to phosphorus, it is only 0.08 equivalent to the daily dose (3 g, 30 mmol) of calcium carbonate. There is a report that oral administration of 1350 mg/day (8 mmol) of chitosan for 12 weeks in HD patients reduced serum total cholesterol level significantly, but did not affect serum phosphorus levels [19]. This suggests that chitosan itself would not be a single player as a phosphorus binder and additional effects are likely to be involved in chitosan-loaded chewing gum.

Second, we expected an add-on effect of chitosan to phosphorus binders. The lack of dose of phosphorus binders, wherein the required dose cannot be administered because of their side effects, would be covered by HS219. However, we should know the expected add-on effect might be less than 10% and its contribution would be limited.

Third, we expected an chewing effect on salivary flow rate. Chewing gum is reported to stimulate salivary flow rate in healthy adults [20,21] and in HD patients [22,23]. Increased salivary flow increases the gut fluid volume and solid (phosphorus binder)-liquid (phosphorus) reaction would be accelerated and result increase the yield of phosphorus trapping. We considered that a chewing time of 30 min was sufficient to compare with the 1-h chewing time in Savica et al.’s study [15]. This is because the peak of chewing-stimulated salivary flow rate has been reported to be approximately 6 ml/min in the first minute and decreases to a plateau of approximately 1 ml/min across the next 15 min in healthy adults [24]. With regard to salivary flow rate in HD patients, two different observations have been made; one is that there was no difference compared with healthy controls [25,26] and the other was that salivary flow rate was less than that in healthy controls [9,27].

We expected that the sum of these three hypotheses described above could explain the rationale of the efficacy of chitosan-loaded chewing gum. However, unfortunately, in our study, the chitosan (40 mg)-loaded chewing gum HS219 did not show any effect of reduction in salivary and serum phosphorus levels. This finding suggests that the chitosan dose was still too low, and that neither an add-on effect nor chewing effect of chitosan-loaded chewing gum reflected in the clinical outcome. Furthermore, HS219 did not result in any biological responses of serum Ca, Ca x phosphorus products, iPTH, whole PTH, or FGF23 compared with placebo. Block et al. reported similar negative results of chitosan (20 mg)-loaded chewing gum by a randomized, placebo-controlled, double-blind study in HD patients [28]. In chronic kidney disease patients, chitosan chewing gum reduced serum phosphorus levels by only 0.14-0.20 mg/dl [28]. These two independent randomized, placebo-controlled, double-blind studies revealed a better understanding of the lack of clinical benefits of chitosan-loaded chewing gum in HD patients. The positive results from the open-label, non-controlled study by Savica et al. [15] were a result of biases, as commented by Oh and Uribarri [29]. Our study suggests that there are pitfalls in non-randomized, open-label clinical studies.

Although Savica et al. [15] reported a good association between salivary and serum phosphorus levels, we did not observe any association between these variables at baseline (n = 63, r = 0.136, p = 0.287). However, mean salivary phosphorus levels in HD patients in our study were approximately 3.5 times higher than those of serum levels. Daily production of saliva in healthy subjects is reported to be 500–1000 ml [10]. Salivary flow rates of HD patients enrolled in our study were higher than 3.5 g/2 min by the Saxon test, which are similar to those of normal subjects. This finding indicates that the supply of phosphorus from saliva to the gut might be 118–240 mg/day, which is a considerable amount, as well as that from food. Although HS219 did not show any efficacy in patients with hyperphosphatemia, salivary phosphorus still remains a potent and novel target of phosphorus restriction.

FGF23 is one of the most recently identified phosphoric factors, which regulate mineral and vitamin D metabolism [30]. Serum iFGF23 levels in HD patients are elevated over a wide range [31], and FGF23 levels in our study were extremely elevated. We observed a good correlation between serum phosphorus and iFGF23 levels at baseline, and between changes in serum phosphorus and FGF23 levels. The lack of effect of HS219 on serum FGF23 levels provides additional evidence that HS219 does not affect phosphorus metabolism in HD patients with hyperphosphatemia.

## Conclusions

Our randomized, placebo-controlled, double-blind study shows that HS219 (chitosan-loaded chewing gum) did not show any add-on effect on salivary and serum phosphorus levels in HD patients with hyperphosphatemia who are treated with either sevelamer hydrochloride or calcium carbonate.

## Abbreviations

ESRD: End-stage renal disease; HD: Hemodialysis; iPTH: intact parathyroid hormone; iFGF23: intact fibroblast growth factor 23.

## Competing interests

This study was funded by KDL Inc., Tokyo, Japan. TA has been a consultant to Kyowa-Hakko Kirin, REATA, Abbvie, and Bayer Japan. TA has also received lecture fees or honoraria from Kyowa-Hakko Kirin, Chugai, Astellas, and Bayer Japan, as well as grant support from Chugai, Bayer Japan, Kyowa-Hakko Kirin, Baxter, and Daiichi-Sankyo. MF has been a consultant for Kyowa-Hakko Kirin, Chugai, Bayer Japan, Novatis, JT, Abbvie, and Astellas. MF has also received lecture fees or honoraria from Kyowa-Hakko Kirin, Chugai, Bayer Japan, and Astellas, as well as grant support from Kyowa-Hakko Kirin and Chugai. The results presented in this paper have not been published previously in whole or in part except in ClinicalTrials. gov. and abstract format.

## Authors’ contributions

TA and MF designed the study, analyzed the results, and drafted the manuscript. YT, YO, Y. Miyauchi, AS, HK, NS, Y. Maeda, Takako S, NM, JN, Toshiaki S, and YA are clinicians, who performed this study at different centers. HH is a biochemist who measured salivary phosphorus levels. SK participated in designing the study and performed the statistical analysis. All authors read and approved the final manuscript.

## Pre-publication history

The pre-publication history for this paper can be accessed here:

http://www.biomedcentral.com/1471-2369/15/98/prepub
